# Engineered nanomaterials released from commercial nano-enabled products affect the morphology and behaviour of the zebrafish (*D. rerio*) embryo

**DOI:** 10.1007/s10646-026-03139-z

**Published:** 2026-08-20

**Authors:** Mbuyiselwa Shadrack Moloi, Raisibe Florence Lehutso, Dana Kühnel, Nils Klüver, Mariana Erasmus, Paul Johan Oberholster, Melusi Thwala

**Affiliations:** 1 Centre for Environmental Management, University of the Free State, Bloemfontein, South Africa; 2 Water Research Centre, Council for Scientific and Industrial Research, Pretoria, South Africa; 3 Department of Ecotoxicology, Helmholtz Centre for Environmental Research – UFZ, Leipzig, Germany; 4 Centre for Mineral Biogeochemistry, University of the Free State, Bloemfontein, South Africa; 5 Science Advisory and Strategic Partnerships, Academy of Science of South Africa, Pretoria, South Africa

**Keywords:** Nano-enabled products, Environmental exposure, Product-released engineered nanomaterials, Ecotoxicity, ZEF, Zebrafish behaviour

## Abstract

The increasing use of nano-enabled products (NEPs) is contributing to nanopollution, with product-released engineered nanomaterials (PR-ENMs) posing varying risks to aquatic organisms. However, data on their environmental biological impacts remain limited. This study investigates the ecotoxicological effects of PR-ENMs, specifically nTiO₂, nZnO, and nAg, released from commercially available NEPs (sunscreens, topical creams, and socks) using zebrafish (*Danio rerio*) embryos as a vertebrate model. PR-ENMs were generated through simulated consumer-use scenarios and characterised for size, charge and elemental composition to determine their physicochemical profiles. The zebrafish embryo toxicity test was employed to evaluate mortality, developmental abnormalities, and behavioural responses, providing a multi-endpoint assessment. PR-ENMs induced concentration- and material-dependent toxic effects. PR-nZnO and PR-nAg notably triggered both morphological deformities and significant alterations in both spontaneous and stimulus-induced movement, suggesting neurodevelopmental disruption. Ion release contributed substantially to toxicity, with ionic and particulate forms eliciting comparable responses. The integration of behavioural and morphological endpoints enabled the early detection of sublethal effects and provided mechanistic insights into PR-ENM toxicity. These results emphasise the need to incorporate early detection strategies in nanomaterial hazard assessment and highlight the ecological relevance of realistic exposure scenarios. Overall, this study advances the understanding of PR-ENMs’ interactions with aquatic biota and supports more predictive and ethically responsible toxicological evaluations. Further studies are, however, recommended to elucidate the effects in complex environmental systems.

**Supplementary Information:**

The online version contains supplementary material available at https://doi.org/10.1007/s10646-026-03139-z.

## Introduction

Engineered nanomaterials (ENMs) are novel entities whose rising anthropogenic utilisation presents increasing potential for negative environmental consequences, a trend that necessitates heightened safety assessment efforts to contain transgression of the planetary boundary for novel entities (Persson et al. 2022). The consumption of nano-enabled products (NEPs) is estimated to reach USD 288 billion by 2030 (Precedence Research, 2021), primarily due to increase in the composites, health, and personal care products that are nano-enabled. Throughout the lifecycle of NEPs, there is potential for environmental release of ENMs (nanopollution) (Caballero-Guzman and Nowack 2016), however, the rate of production and release dynamics outstrip the global capacity to adequately assess theirsafety implications.

The occurrence, environmental release and accumulation of ENMs, coupled with concerns on their potential health and environmental impact, have triggered global effects assessment in aquatic environments. Ecotoxicity investigations thus far point to potential toxicity effects of ENMs, the large body of evidence derived from studies of pristine ENMs (Baun et al. 2008; Blinova et al. 2010; Park et al. 2014; Thiruvengadam et al. 2024). However, there are limitations in drawing robust environmental risk conclusions based solely on data from pristine ENMs, as such data do not fully account for real-world scenarios (Carboni et al. 2023). For instance, detected environmental concentrations of ENMs tend be lower than the respective predicted no-effect concentrations obtained from pristine forms (Huang et al. 2024). For enhanced environmental realism, it is essential to increase safety assessment effort using ENMs that are most likely be detected in the environment, that is, ‘form-specific’ product-released ENMs (PR-ENMs). However, this approach is still in its infancy stage (Baek et al. 2017; Fastelli and Renzi 2019; Hong et al. 2021).

Most nanotoxicology studies have primarily focused on aquatic micro- and macroinvertebrates (Arora et al. 2012; Book et al. 2019; Ma et al. 2013). However, studies on the effects of ENMs using the zebrafish (*Danio rerio*) embryo are emerging, with effects including reduced swimming ability (T. H. Chen et al. 2011), morphological alterations (Cunha and De Brito-Gitirana 2020a), and mortality (Sezer et al. 2023) reported. For ‘form-specific’ PR-ENMs, studies are so far very rare, with only two peer-reviewed reports identified (search conducted up to July 2025). These studies investigated the effects of nAg released from textile products and reported contradictory findings (Gao et al. 2015; Reed et al. 2016). Using the standard zebrafish embryo test, Gao et al. (2015) observed that PR-nAg inhibited hatching and normal embryo development and induced mortality after 24 h (LC_50_ range of 0.26–0.4 mg/L), whereas Reed et al. (2016) reported no observable effects on *D. rerio*. The exposure concentrations used in both studies were comparable and represented ENM amounts released from a single wash of the NEP during product usage. However, the NEP matrices were different, and thus, it can be expected that “other” components associated with PR-ENMs varied, a phenomenon that further complicates realistic risk assessment efforts. Notwithstanding these findings, the data presented herewith shows a notable data gap on the understanding the PR-ENMs’ toxicity and underlying mechanisms in *D. rerio* at both lethal and sublethal levels. Although in silico models are increasingly used to address such data gaps, they require extensive bioactivity datasets (Leuthold et al. 2019), which currently lack for PR-ENMs.

The current study investigated the acute effects of PR-ENMs on *D. rerio* embryos, with mortality, morphological effects, and behaviour as endpoints. Given the limited data on PR-ENMs and *D. rerio* interactions, this study investigated both lethal and sublethal effects following exposure to four different PR-ENMs, thereby making an essential contribution to addressing data paucity.

## Materials and methods

### Physicochemical properties of PR-ENMs

ENMs released from four NEPs were obtained using simplified methods previously described by Lehutso and Thwala (Lehutso and Thwala 2021) and presented in Moloi et al. (2025). Briefly, engineered nanomaterials (ENMs) from solid samples (SS1, SS2, TC) and fabrics (SC) were released using modified agitation-based extraction methods. For SS1 and SS2, 2 g of each material was mixed with 175 mL of Milli-Q water and agitated at 400 rpm for 48 h at ambient temperature, followed by 48 h settling to allow phase separation (total release time of 96 h). A 150 mL supernatant was carefully collected for ENMs characterization and ecotoxicity assessment. For TC, 2 g of materials was agitated in 175 mL of Milli-Q water at 400 rpm for 24 h at 150 °C to account for its non-polar nature, after which the suspension was immediately filtered for ecotoxicity assessment. For SC, pre-confirmed ENM-containing sections were immersed in 150 mL Milli-Q water with two glass marbles to simulate mechanical stress. Samples were agitated at 200 rpm for 48 h at 40 °C, and supernatants were collected for characterization and ecotoxicity assessment. All samples were filtered sequentially at 0.45 and 0.2 μm to remove, as much as possible, the organic matrix and any suspended solid from the nano-fraction of the target PR-ENMs samples. Although the filtration removes majority of the organic matrix from the nano-fraction samples, PR-ENMs remain associated with the residual organic material matrix that escapes filtration. Hence, all exposures were conducted using ‘form-specific’ PR-ENMs, i.e. PR-ENMs still associated with the organic matrix of the product. Ions were recovered from TC and SC through sequential filtration. Briefly, the release samples were sequentially filtered using Amicon^®^ Ultra-15 30 K centrifugal filters (30000 MWCO) (Merck, Johannesburg, South Africa), followed by further centrifugation using Amicon^®^ Ultra-15 3 K centrifugal filter devices (3000 MWCO) (Merck, Johannesburg, South Africa) for 5 min at 10 000 rpm for each filtration step. The released ions (filtrates from the 3 K centrifugal filter device) were used for exposure assessment.

The PR-ENMs varied in their physicochemical properties (Table 1); extensive details of the release methods and physicochemical properties of the PR-ENMs utilised in this study are described in Moloi et al. ( 2025). No targeted analysis of residual organic constituents in the PR-ENMs was performed. Although such materials may contain trace organic compounds derived from their source products, the products from which the PR-ENMs were obtained were highly heterogeneous in composition and formulation. Consequently, the potential spectrum of organic constituents was too broad and variable to enable a meaningful targeted analytical approach.

**Table 1:** The physico-chemical properties of the assessed PR-ENMs

NEP	Sample ID	Identified ENMs	Shape	Size (nm)	Zeta potential (mV)	Other associated materials^a^
Sunscreen 1	SS1	nTiO_2_	Angular	39.5 ± 1.5 × 9.2 ± 0.3	-94.5 ± 1.9	Si, Al
Sunscreen 2	SS2	nTiO_2_	Needle-shaped	33.1 ± 1.2 × 11.6 ± 0.5	-78.3 ± 0.9	Si, Al
Topical Cream	TC	nZnO	Angular	109.6 ± 6.6 × 55.7 ± 3.9	-15.5 ± 1.9	Si, Zn^2+^
Sock	SC	nAg	Near-spherical	58.8 ± 4.3 × 39.4 ± 3.8	-50.1 ± 1.7	Si, Ag^+^

^a^Other materials were quantified in the matrix, which were either the associated ENMs coating or ions released by the PR-ENMs

### Zebrafish culture embryo production

Wild-type in-house strains of the zebrafish (*Danio rerio*) were used to assess the impact of ENMs released from commercial NEPs. Fish were cultured at 26 ± 1 °C at a 14:10-hour light: dark cycle in a recirculating tank system and used according to German and European animal protection standards and approved by the Government of Saxony, Landesdirektion Leipzig, Germany (Aktenzeichen 75–9185.64).

To harvest the eggs, trays with artificial plants were placed in tanks with male and female fish in the evening. Spawning took place shortly after the lights turned on in the morning. Eggs were collected, and fertilised eggs were sorted and cultured from 2 h post-fertilisation (hpf) in control embryo medium until exposure at 26–28 °C.

### Fish embryo ecotoxicity assay

OECD Test guideline 236 (Fish embryo acute toxicity test) (OECD, 2013) and the ISO water quality for freshwater fish semi-static method (ISO 12890: 1999) (ISO, 1999) were followed in all tests. Exposure concentrations were prepared by serial dilution of the release media, with nominal concentrations of 0–1.82 mg/L based on metal concentrations. The PR-ENMs investigated for toxicity included nTiO_2_ from sunscreens (SS1 and SS2), nAg from textiles (textile, SC) and nZnO from baby cream (TC). To determine mixture toxicity in ion-releasing fraction (from TC and SC, 0.00015–1.816 mg/L) in all the ecotoxicity bioassays, the response addition model (RA) (Ye et al. 2018) was used following Eq. 1:

1 $$\mathrm{E}_{\mathrm{Total}}=(1-\mathrm{E}_{\mathrm{ion}})(1-\mathrm{E}_{\mathrm{particle}})$$

where, E_Total_ and E_ion_ represent the toxicity caused by the PR-ENMs suspensions and their corresponding released ions (scaled from 0 to 1). E_Total_ and E_ion_ were quantified by the toxicity testing. Effects caused by the particles (E_particle_) were directly calculated by the RA model.

The 96 h acute fish embryo test was used to investigate the effects of ENMs and their ionic forms released from SS1, SS2, SC and TC. Preliminary tests showed that UW (PR-nTiO_2_) did not induce any acute effects. All exposure concentrations and media were prepared using ISO water at pH 7.4 (ISO 12890: 1999). Fish eggs (≥ 2 hpf) (OBI-WIK strain, F3 generation) obtained from broodstock cultured under 14:10 h (light: dark) photoperiod in 120 L aquaria, were exposed to TC PR-nZnO ( 0.02–1.82 mg/L), SC PR-nAg and PR-Ag^+^ ( 0.0005–0.008 mg/L) and SS1 PR-nTiO_2_ and SS2 PR-nTiO_2_ ( 0.0002–0.47 mg/L) over 96 h. Mortality and malformations (as stipulated in the OECD Test 236 guidelines) were used as main endpoints. In addition, behavioural endpoints were assessed. A single embryo was exposed in 400 µL of the exposure medium, and 12 replicates were used per concentration in a 96-square-well plate. The plates were incubated at 28 ℃ throughout the exposure period and assessed every 24 h for mortality and malformations. For quality assurance, the test was considered valid if the mortality in the control was ≤ 10%.

### Morphological assessment

The morphological effects of PR-ENMs were assessed using the Vertebrate Automated Screening Technology (VAST) BioImager Platform (Union Biometrica Inc., Massachusetts, USA), coupled with a Leica Fluorescence Microscope (Leica Microsystems, Wetzlar, Germany) as described by Bittner et al. (2018). The 96-h-old larvae were anaesthetized with tricaine solution (0.3 mg/mL final concentration, pH adjusted to 7.1–7.3) and sampled into a capillary for automated positioning. The larvae were imaged in lateral, dorsal and ventral orientations. The images taken by the VAST system were further utilised for phenotype evaluation, specifically targeting malformation of the head, indicated by reduced head and eye size (mhe), oedema of yolk and pericard (EdY/EdP), curved spine (axis), and the swim bladder. Image analysis was performed using an amended version (1.70, available via https://codebase.helmholtz.cloud/ufz/tb3-cite/biotox/FishInspector) of the FishInspector imaging software (Teixidó et al. 2022).

### Fish behavioural assessment

#### Spontaneous tail coiling (STC)

Fish embryos were exposed to different PR-ENMs as described in Sect.  2.3. d STC analysis was conducted by using a gentle touching stimulus after 24 h (Ogungbemi et al. 2020), in addition to assessing mortality and malformations. Plates were videotaped for 60 s (2 frames per second) each for STC using an Olympus DP21 video camera (Hamburg, Germany) mounted on the Olympus SZX7 stereomicroscope (Hamburg, Germany) with 0.8× magnification. The plates were analysed against a black background and dark-field transmitted light at the base of the microscope with an ISO speed of 400. The shutter speed was 1/80, and the image size was 400 × 300 pixels. The obtained videos were analysed using the KNIME^®^ Analytical Platform (Berthold et al. 2009). Wells in which mortality was observed prior to video recording were excluded from further analysis.

#### Photomotor response (PMR)

The PMR was assessed 6 h after STC (30 hpf) following the same procedure as described for STC, with slight modification to the protocol of Saint-Amant and Drapeau (Saint-Amant and Drapeau 1998). Briefly, after 30 h, a photic stimulus was applied for 60 s. The excitation phase (5–7 s) and refractory period (15 s) were recorded by measuring embryonic movement tracked using the ZebraBox video tracking system (Viewpoint, Lyon, France) at 26 ± 1 °C. The videos were analysed using the KNIME^®^ Analytical Platform (Berthold et al. 2009).

#### Locomotor response (LMR)

For the LMR, exposure was carried out as described in Sect.  2.3. After 96 h, embryo movement was analysed using a modified protocol described in Klüver et al. (Klüver et al. 2015). Plates were first assessed for mortality and malformations before movement analysis and recorded accordingly. Non-hatched embryos were dechorionated manually using a Pasteur pipette prior to locomotion analysis. The movement of the embryos in the wells was tracked using the ZebraBox video tracking system (Viewpoint, Lyon, France). Embryonic movement videos were recorded using a 25 frame/s infrared camera for 20 min (5 min light, 10 min dark, 5 min light). Locomotion during the dark phase was then used to calculate the differences between control and treated groups. All of the wells in which mortality was observed prior to LMR analysis were excluded from further analysis.

### Data analysis

Concentration–response relationships were modelled using non-linear regression based on a four-parameter logistic (4PL) function to determine LC₅₀ values. Parameter estimation was performed by least-squares optimisation in XLSTAT Life Sciences software (Addinsoft Inc., Paris, France). Experiments were conducted with seven replicates per treatment across seven exposure concentrations. Differences in mortality among concentration groups were assessed using one-way analysis of variance (ANOVA), followed by an appropriate post hoc multiple comparison test to identify statistically significant pairwise differences. The statistical significance was accepted at *p* ≤ 0.05 (confidence interval = 95%).

## Results and discussion

### Ecotoxicity effects of PR-ENMs on *D. rerio*

**Fig. 1:**
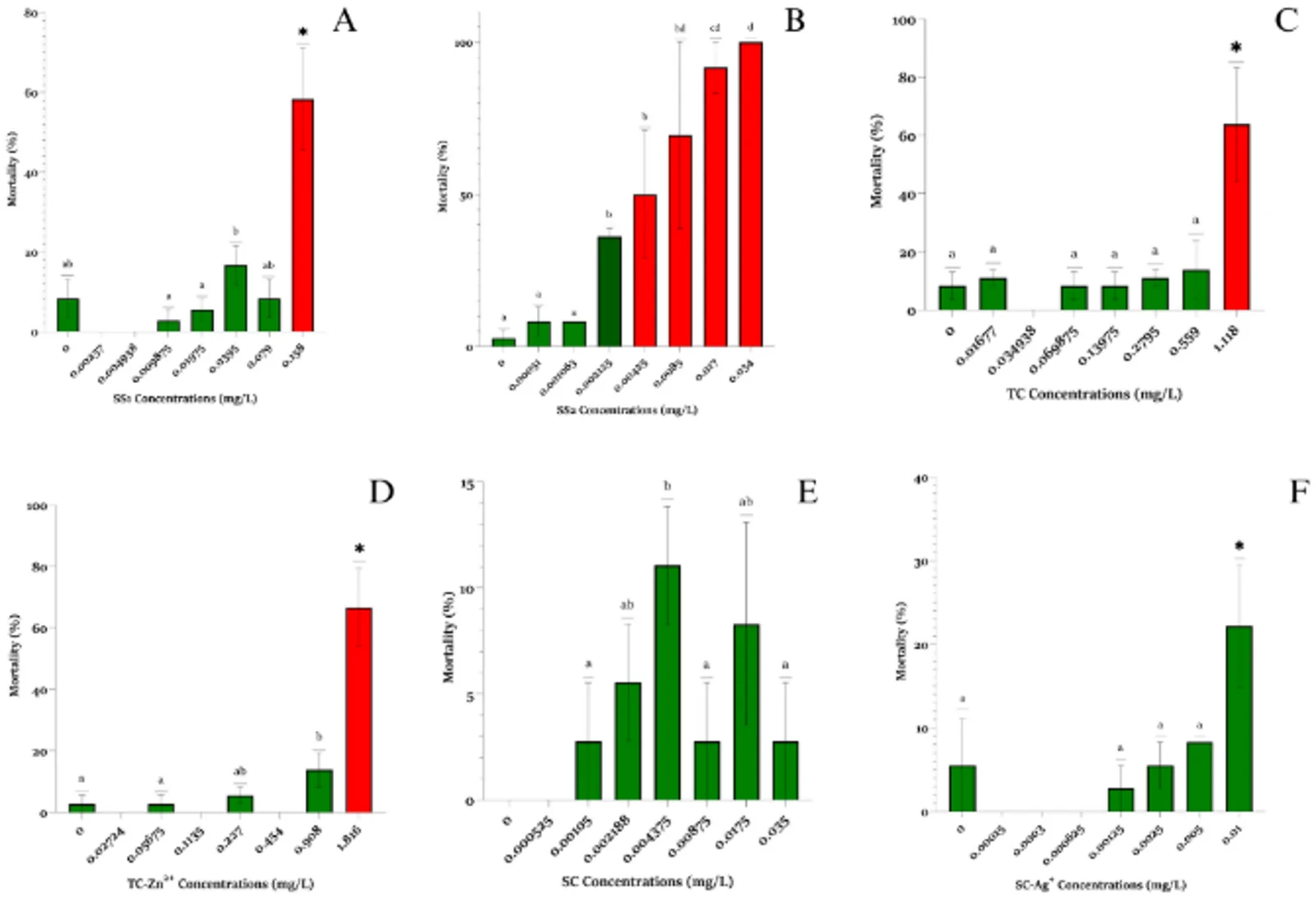
The average mortality of *D. rerio* induced by SS1 PR-nTiO_2_ (**A**), SS2 PR-nTiO_2_(**B**), TC PR-nZnO (**C**), TC PR-Zn^2+^ (**D**), from SC PR-nAg (**E**), and SC PR-Ag^+^ (**F**) after a 96-hour exposure. The letters a, b, c, and d represent statistical differences within groups. The asterisk (*) represents statistically significant data, at *p* ≤ 0.05

*D. rerio* embryos were affected differently by PR-ENMs, with responses ranging from no effects to mortality (Fig. 1). SS1 PR-nTiO_2_ induced lower mortality on *D. rerio* embryos (LC_50_=0.66 mg/L) relative to SS2 PR-nTiO_2_ which resulted in a markedly lower LC_50_ of 0.005 mg/L (average mortality = 45.82%) (Fig. 1A, B). The difference in toxicological potential between the two PR-nTiO_2_ materials (SS1 and SS2) may be linked to the differing shape and size of the ENMs; the SS1 were needle-shaped whereas SS2 were angular, with average ENMs’ sizes of 18 × 75 (*w×l*) and 52 × 69 (*w×l*) nm, respectively. The shape of nTiO_2_ has been shown to influence their toxicity to zebrafish embryos (Allegri et al. 2016; Musial et al. 2020); this applies to other ENM forms. For instance, nTiO_2_ nanofibers were shown to induce higher toxicity by reducing cell viability, altering cell morphology, inducing oxidative stress, and generating ROS in cell cultures (mouse peritoneal monocyte-macrophage cell line Raw 264.7 and human alveolar carcinoma epithelial cell line A549), whereas angular nTiO_2_ only induced mild effects (Allegri et al. 2016). Although the toxicity of nTiO_2_ on *D. rerio* embryos survival has been reported previously (Cunha and De Brito-Gitirana 2020b; Fouqueray et al., 2012; Osborne et al. 2013; Prakash et al. 2019), to the best of the authors’ knowledge, this is the first report on the effects of sunscreen-released nTiO_2_ on *D. rerio*.

TC PR-nZnO and TC Zn^2+^ induced mild (LC_50_ = 3.38 mg/L) and higher (LC_50_ = 1.01 mg/L) effects, respectively, with average mortalities of 63.87% and 66.63% at the highest exposure concentrations of 1.11 mg/L and 1.81 mg/L after 96 h (Figs. 14 C, D). There was no significant difference (*p* = 0.53) between the toxicity of PR-nZnO and TC Zn^2+,^ even though TC Zn^2+^ appeared relatively more toxic. Both TC PR-nZnO and TC Zn^2+^ contributed to the observed effects in *D. rerio* embryos; similar findings have been reported elsewhere (Ribeiro et al. 2014; Wang et al. 2016). Similar to sunscreen-released ENMs, this represents the first report on the effects of topical cream-released nZnO on fish embryos. SC PR-nAg caused effects in *D. rerio* embryos (LC_50_ = 0.001 mg/L), although negligible mortality was observed across concentrations, whereas SC Ag^+^ induced notable mortality in a concentration-dependent manner (Fig. 1E and F). However, LC_50_ for SC Ag^+^ could not be determined. The toxicity effects induced by SC-nAg and SC-Ag^+^ on *D. rerio* embryos were not significantly different (*p* = 0.646). Previous studies (Reed et al. 2016) have reported that sock-derived PR-nAg considerably affected *D. rerio* embryo development and induced mortality (LC_50_ of 0.26–0.4 mg/L). In another study (Gao et al. 2015), no effects were recorded for *D. rerio* embryos after a 24 h exposure to sock PR-nAg. In the present study, mortality was observed; however, it was not consistently concentration-dependent in all cases (Fig. 1E). Notwithstanding these observations, the effects on survival warrant further investigation into sub-lethal endpoints which may later affect the survival of the organisms (Horie et al. 2017). Overall, PR-ENMs induced varying effects on the survival of D. rerio embryos, with PR-nAg and PR-nTiO₂ inducing the highest and lowest toxicity, respectively. Notably, a white layer resulting from PR-ENMs was observed to surround the chorion, particularly in embryos that failed to hatch and subsequently died. Although this was not part of the current investigation, previous studies suggest that ENMs may delay or prevent successful hatching of the embryo, thereby contributing to mortality (Pereira et al. 2023a). While the chorion serves as an effective protection layer, its pores (0.7–0.9 μm) may permit passage or internation of PR-ENMs which are smaller (0.039–0.109 μm) (Pereira et al. 2023b). Accumulation of ENM aggregates and associated product matrix on the chorion surface may clog these pores, potentially leading to oxygen depletion and impaired embryo survival. Direct uptake via ingestion is not relevant since the juveniles don’t feed, but absorption remains possible. Although PR-ENMs clearly induced effects, the role of the chorion and interaction with free-swimming juveniles warrant further investigation.

### Effects of PR-ENMs on *D. rerio* morphology

PR-ENMs induced morphological changes in *D. rerio* (SI, Figures S1–S6). In all instances, the notochord curvature was the most common effect, whereas the was in the swim bladder showed the least morphological change relative to the control. The overall toxicity trend in descending order was SS1 > TC = SC≥SS2.

SS1 PR-nTiO_2_ induced notochord curvature across all exposure concentrations (0.01–0.47 mg/L). Similar effects were observed following exposure to SS2 PR-nTiO_2_, TC PR-nZnO, and TC PR-Zn^2+^. The highest concentration (0.03 mg/L) of SC PR-nAg induced notochord curvature, while slightly affecting the otolith formation at the lower concentrations (1.050 × 10^− 3^–0.018 mg/L). The effects observed for SC PR-Ag^+^ exposure were less pronounced. Scoliosis, as evidenced by the notochord curvature, can impair swimming ability (Von Hellfeld et al. 2020), potentially altering organism’s foraging efficiency and predator avoidance (Jablonski et al. 2022). Exposure to SS1 PR-nTiO_2_ increased the pericardial size in a concentration-dependent trend, leading to pericardial oedema. The opposite effects were observed for SC PR-Ag^+^, where the pericardium size was considerably reduced. Mild pericardial effects were observed following exposure to SS2 PR-nTiO_2_, TC PR-nZnO, TC PR-Zn^2+^, and SC PR-nAg. Oedema affects the heart rate (not assessed in the current study) and consequentially survival in *D. rerio* (Ames et al. 2022; Liu et al. 2022).

Alterations in pigmentation were observed after exposure to SS1 PR-nTiO_2,_ TC PR-nZnO, SC PR-nAg, and SC PR-Ag^+^ (Figure S2–S8). In SS1 PR-nTiO_2,_ pigmentation was reduced considerably from the lowest concentrations, with the effects rising with the increasing exposure concentration. Hypopigmentation was also evident in TC PR-nZnO exposure, whereas TC PR-Zn^2+^ exposure induced mild effects. In contrast, SC PR-nAg caused hyperpigmentation at lower concentrations (1.05–8.75 × 10^− 3^ mg/L), while effects from SC PR-Ag^+^ exposure were minimal. Pigmentation plays a critical in camouflage and avoidance of predators (Morash et al. 2022).

Swim bladder size increased considerably at 7.11 × 10^− 4^–0.12 mg/L but was unaffected at the highest concentrations (0.24–0.47 mg/L) in SS1 PR-nTiO_2_ exposures. The swim bladder controls the organism’s buoyancy (Jablonski et al. 2022). Otolith formation was also affected: SS1 PR-nTiO_2_ induced variable effects across concentrations; in SS2 PR-nTiO_2_ increased the mouth tip-otolith distance, TC PR-nZnO reduced otolith size in a concentration-dependent manner); and TC PR-Zn^2+^ increased the mouth tip otolith angle). Otoliths are essential for detecting linear accelerations and gravity, thereby maintaining balance (Kang et al. 2008). The interocular distance was also adversely affected, decreasing with increasing concentrations. Such changes may impair vision-guided behaviours in the larvae and adults (LeFauve and Connaughton 2017).

Overall, the morphological abnormalities observed during early stages of development may compromise survival to adulthood. Effects such as scoliosis, pericardial oedema, and altered pigmentation can impair physiological processes, behaviour and overall survival. The current findings are evidence of PR-ENMs’ morphological toxicity in *D. rerio* larvae, with potential implications for survival and ecological function.

### Effects of PR-ENMs on the behavioural endpoints of *D. rerio* embryo and larvae

STC and PMR are commonly used as behavioural endpoints associated with chemical developmental neurotoxicity (DNT) (Marcato et al. 2015; Younes et al. 2018). Pollutants such as organophosphates and pharmaceuticals have been shown to cause DNT in zebrafish (Pham et al. 2016; Zindler et al. 2019). Changes in STC activity, whether stimulation or inhibition, indicate potential adverse effects on the nervous system (Younes et al. 2018). LMR on the other hand exhibits increased sensitivity beyond acute tests, particularly for neuroactive compounds (Leuthold et al. 2019).

#### Spontaneous tail coiling (STC)

**Fig. 2:**
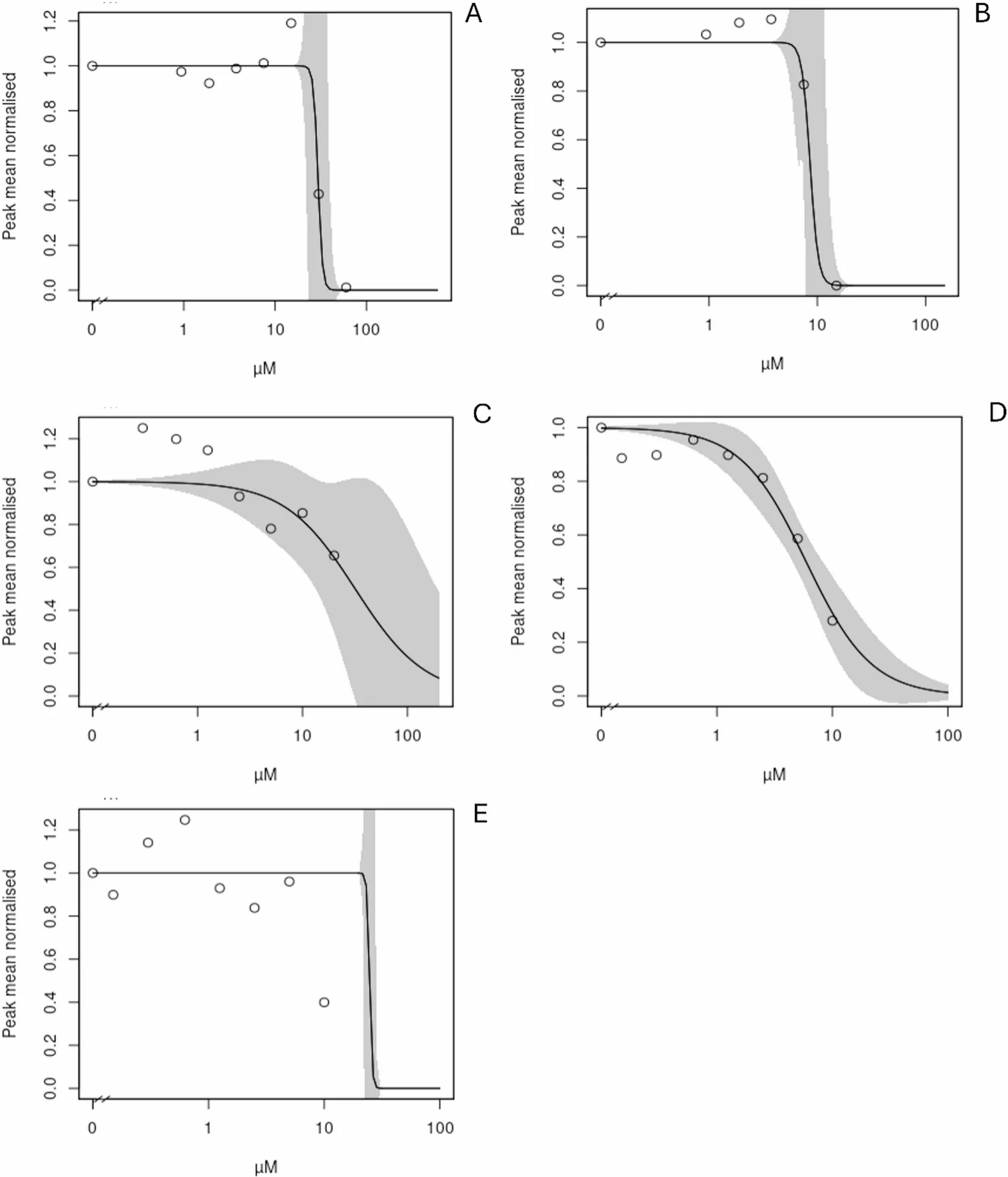
The STC activity of *D. rerio* embryos exposed to PR-ENMs from SS1 PR-nTiO_2_ (**A**), SS2 PR-nTiO_2_ (**B**), TC PR-nZnO (**C**), SC PR-nAg (**D**) and SC PR-Ag^+^ (**E**) after the 24 h exposure period. The STC measurement for TC Zn^2+^ was unsuccessful. The 0-100% correspond to 0–0.16 mg/L, 0–0.03 mg/L, 0–1.81 mg/L, 0–0.03 mg/L and 0–0.01 mg/L for SS1 PR-nTiO_2_, SS2 PR-nTiO_2,_ TC PR-nZnO, SC PR-nAg, and SC PR-Ag^+^, respectively. The ‘peak mean normalised’ in the y-axis indicates the average spontaneous tail coils over one minute

The STC activity of *D. rerio* embryos was inhibited by PR-ENMs after 24 h exposure to SS1 and SS2 PR-nTiO_2_ at the highest concentration (0.034–0.158 mg/L), whereas the lowest concentration (1.0625 × 10^− 3^–4.94 × 10^− 3^ mg/L) caused no observable effects (Fig. 2A and B). TC PR-nZnO progressively inhibited STC activity in a concentration-dependent manner (Fig. 2C). The STC assessment for Zn^2+^ that is omnipresent with PR-nZnO was unsuccessful, probably due to the low observed toxicity of the released Zn^2+^. Similarly, SC PR-nAg hindered STC activity with increasing exposure concentrations (Fig. 2D). In contrast, SC Ag^+^ stimulated STC activity at lower concentrations (1.05 × 10^− 3^–2.18 × 10^− 3^ mg/L), while severe inhibition was evident at higher exposure concentrations (0.03 mg/L) (Fig. 2E).

In the current study, STC activity was both inhibited and stimulated under different exposure conditions, indicating the potential neurotoxicity of the assessed PR-ENMs. A reduction in the STC activity may indicate structural or functional impairment in muscle development (Ogungbemi et al. 2021). The hyperactivity observed in SC PR-Ag^+^ may reflect epileptic-like movements (Younes et al. 2018).

#### Photomotor response (PMR)

**Fig. 3:**
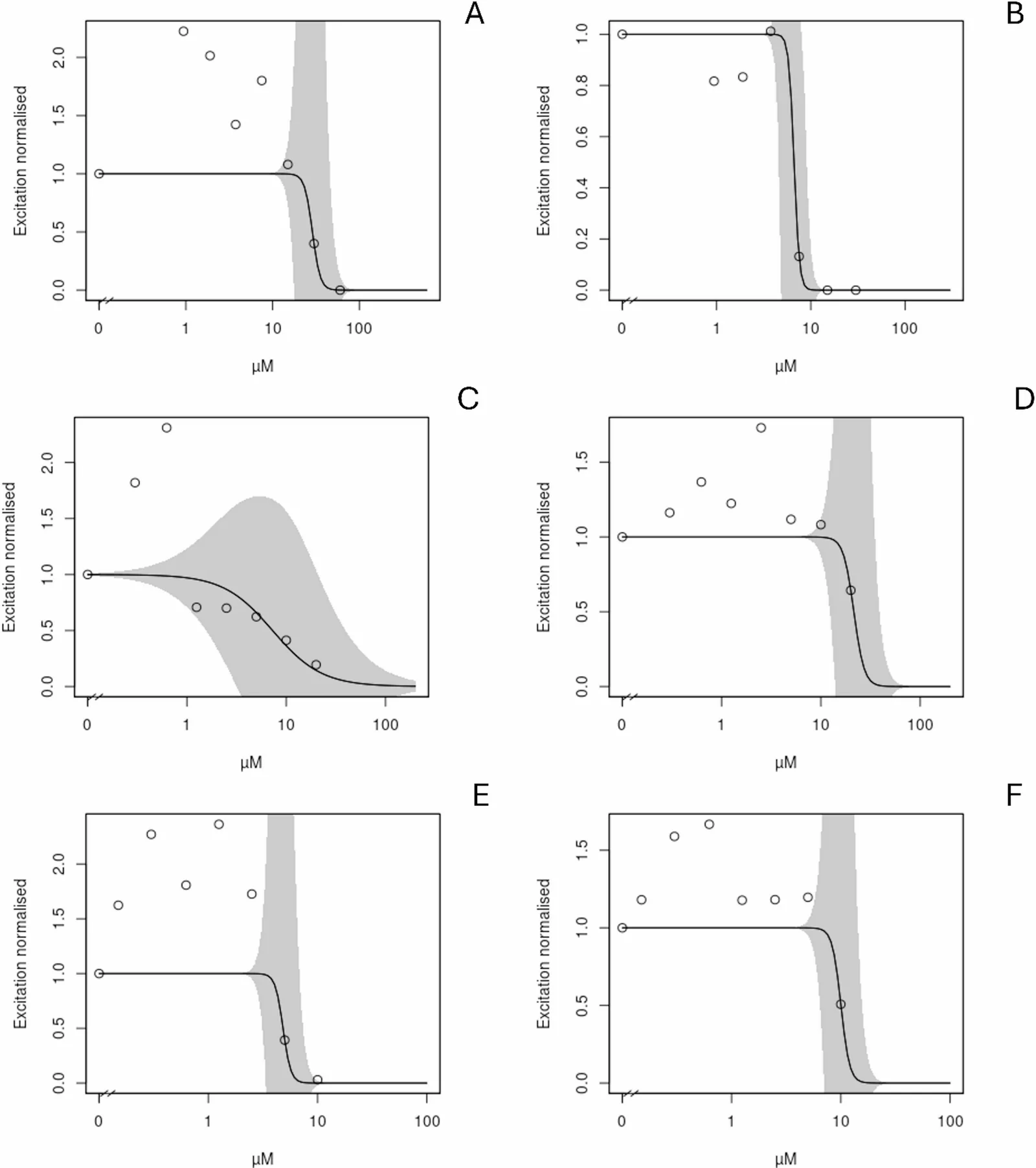
The PMR activity of the *D. rerio* embryo exposed to PR-ENMs SS1 PR-nTiO_2_ (**A**), SS2 PR-nTiO_2_ (**B**), TC PR-nZnO (**C**), TC Zn^2+^ (**D**), SC PR-nAg (**E**) and SC PR-Ag^+^ (**F**) after a 30-h exposure. The 0–100% correspond to 0–0.158 mg/L, 0–0.034 mg/L, 0–1.118 mg/L, 0–1.816 mg/L, 0–0.035 mg/L and 0–0.01 mg/L for SS1 PR-nTiO_2_, SS2 PR-nTiO_2,_ TC PR-nZnO, TC PR-Zn^2+^, SC PR-nAg, and SC PR-Ag^+^, respectively. The ‘excitation normalised’ in the y-axis indicates the average motor response to light stimulus over one minute

The lowest concentration (0.0049 mg/L) of SS1 PR-nTiO_2_ caused no observable changes, whereas the highest concentration (0.16 mg/L) inhibited PMR activity in *D. rerio* embryos (Fig. 3A). Contrary to SS1 PR-nTiO_2_, SS2 PR-nTiO_2_ inhibited PMR activity across all exposure concentrations, with complete inhibition recorded at the highest concentration (0.03 mg/L) (Fig. 3B).

For TC PR-nZnO exposures, *D. rerio* embryos’ PMR activities were stimulated at the lowest exposure concentrations (0.03–0.07 mg/L) and gradually inhibited with increasing exposure concentrations (Fig. 3C). In contrast, TC PR-Zn^2+^ induced mild negative effects: PMR activity was stimulated at lower exposure concentrations (0.06–0.23 mg/L) and only inhibited at the two highest exposure concentrations (0.90 and 1.81 mg/L) (Fig. 3D). For textile-released nAg, both the particulate (Fig. 3E) and ionic Ag (Fig. 3F) forms stimulated PMR at lower concentrations (3 × 10^− 4^–4.38 × 10^− 3^ mg/L), with inhibition only observed at the highest concentrations (0.01–0.03 mg/L).

PMR activity is indicative of nervous system function and occurs independently of eye movement (Marcato et al. 2015). Similar to STC activity, neurotoxic materials can alter this response. PMR activity was generally stimulated by PR-ENMs, even at the low exposure concentrations, except for SS2 PR-nTiO_2_. At higher concentrations, PR-ENMs inhibited the PMR activity. Although the mode of action varies based on type of ENMs, the data illustrate that all tested PR-ENMs affected PMR activity in *D. rerio* embryos. Together with STC results, the findings indicate that the assessed PR-ENMs have the potential to induce developmental neurotoxicity (DNT) in *D. rerio* embryos to varying degrees, potentially interfering with neurotransmission processes (De Oliveira et al. 2021). Disruption of neural development may ultimately result in developmental malformations (Zindler et al. 2019).

DNT is well established for organic pollutants (Carbaugh et al. 2020; Ortmann 2021; Sun et al. 2016) and, to a lesser extent for some metals (Olivares et al. 2016; Tu et al. 2017) and ENMs (Chen et al. 2014; Pham et al. 2016). To the authors’ best knowledge, this is the first report of the potential of PR-ENMs to induce DNT. However, other components of the product may contribute to the observed effects, as the release procedure may not eliminate trace organic remnants of the NEPs. Furthermore, more specific mechanistic bioassays are required to definitively evidence DNT.

#### Locomotor response (LMR)

**Fig. 4:**
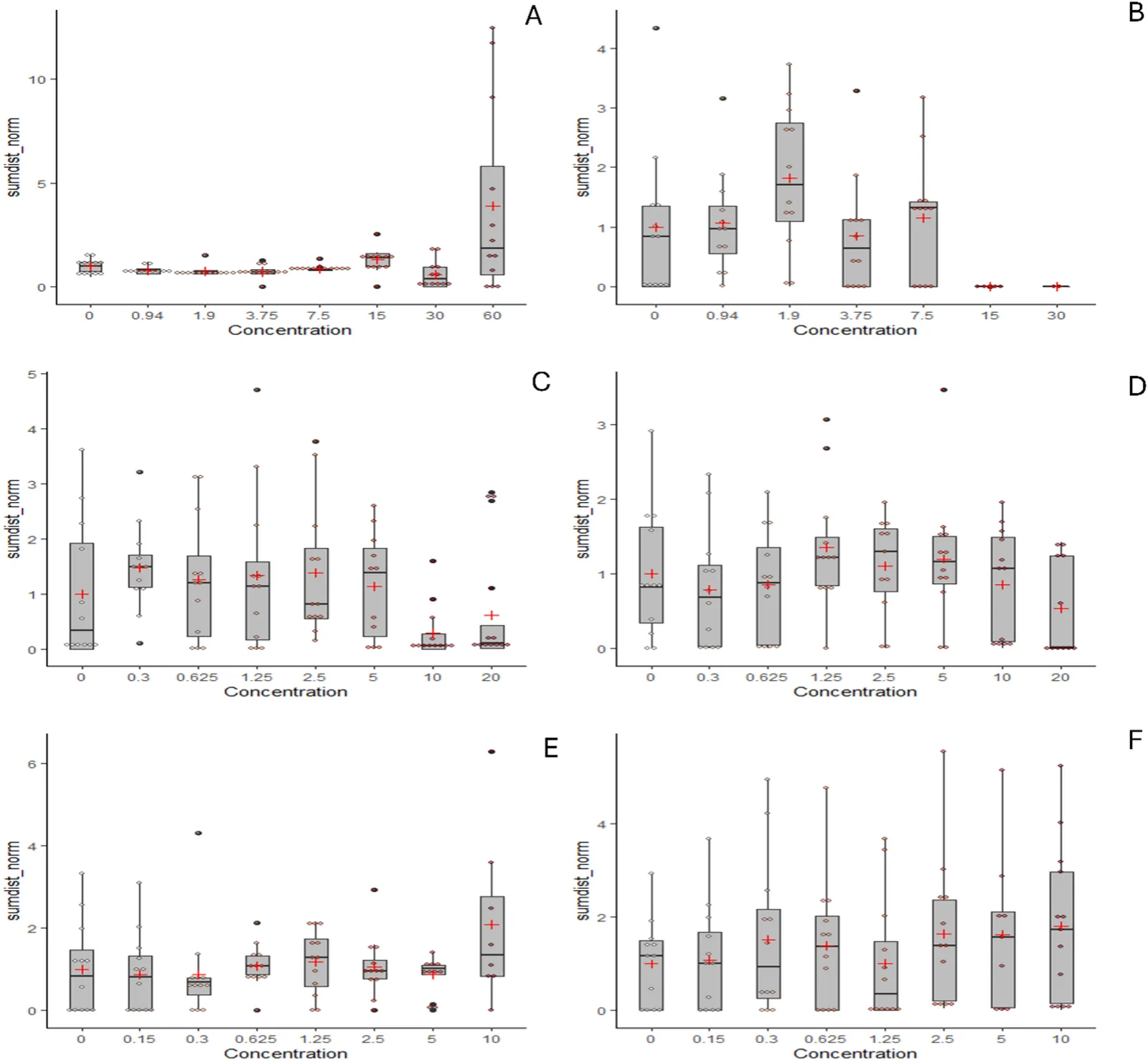
The LMR activity of *D. rerio larvae* exposed to SS1 PR-nTiO_2_ (**A**), SS2 PR-nTiO_2_ (**B**), TC PR-nZnO (**C**), TC Zn^2+^ (**D**), SC PR-nAg (**E**), and SC Ag^+^ (**F**) after a 96-hour exposure period. The 0–100% correspond to 0–0.158 mg/L, 0–0.034 mg/L, 0–1.118 mg/L, 0–1.816 mg/L, 0–0.035 mg/L and 0–0.01 mg/L for SS1 PR-nTiO_2_, SS2 PR-nTiO_2,_ TC PR-nZnO, TC PR-Zn^2+^, SC PR-nAg, and SC PR-Ag^+^, respectively. The y-axis indicates the average distance moved per minute

PR-ENMs affected the locomotor activity of the *D. rerio* larvae after a 96 h (Fig. 4). SS1 PR-nTiO_2_ partially suppressed locomotor activity and only induced rapid hyperactivity (60%) only at the highest concentrations (Fig. 4A). In contrast, the LMR activity was suppressed (hypoactivity) at the two highest concentrations (0.01–0.03 mg/L) of SS2 PR-nTiO_2_ (Fig. 4B). Similarly, LMR activity was suppressed at the two highest concentrations (0.56–1.18 mg/L) for TC PR-nZnO (Fig. 4C). In contrast, TC Zn^2+^ did not induce considerable effects relative to particulate Zn (Fig. 4D), although TC Zn^2+^ slight reduction in LMR activity was observed at at the highest concentrations (1.82 mg/L).

SC PR-nAg mildly affected locomotor activity only at 0.0003–0.0018 mg/L (Fig. 4E). Hyperactivity was primarily induced at the highest concentrations (0.03 mg/L) of SC-nAg, suggesting that high PR-ENM concentrations trigger increased locomotor activity. Random hyperactivity across exposure concentrations was observed for SC-Ag^+^ (Fig. 4F).

Changes in locomotor activity can affect an organism’s physiological capacity to forage, participate in courtship displays, and predation avoidance (Bachour et al. 2020; Xia et al. 2010).

Similar hypoactivity responses to SS1 PR-nTiO_2_, SS PR-nTiO_2_, and SC PR-nZnO exposure have been reported for *D. rerio* larvae exposed to high concentrations of water pollutants (e.g., pharmaceuticals, heavy metals) (Bachour et al. 2020; Tu et al. 2017; Xia et al. 2010). Studies on influence of ENMs on fish behaviour remain limited. Pristine nZnO has been reported to induce hyperactivity in *D. rerio* larvae at 5–10 mg/L concentrations (Chen et al. 2014). The data presented herein provide initial evidence of the adverse effects of PR-ENMs on the locomotor activity of *D. rerio*.

## Conclusions

The current study investigated the effects of PR-ENMs on the zebrafish embryo as a surrogate for the aquatic ecosystem. The PR-ENMs of different types and morphologies induced effects to varying extents. The highest mortality was observed for PR-nTiO2 from SS2, while the least mortality was observed for PR-nAg from SC. The overall toxicity ranking was SS2 > SS1 > TC > SC. The ionic forms derived from PR-nZnO and PR-nAg induced toxicity comparable to their particulate counterparts and, with no statistical differences observed. All PR-ENMs significantly effected the morphology and behavior of *D. rerio*, indicating impact on both physical and neurological development.

Overall, the study illustrated the potential risk posed by PR-ENMs to the zebrafish development. Although zebrafish serve a model organism under controlled environment, effects observed during developmental stages provide key insight into the potential risks to fish populations in the aquatic ecosystems. Impairments in the morphological and neurological development may compromise survival of the organism. Thus, these sublethal effects are critical for understanding immediate acute impacts. In light of the current findings, further research targeting adult fish and more complex systems is recommended to better elucidate long-term and population-level impacts in. Such evidence will supports efforts to guide the design of environmentally safer NEPs.

## Supplementary Information

Below is the link to the electronic supplementary material.

Supplementary Material 1

## Data Availability

No datasets were generated or analysed during the current study.
