# Supplementary material for: Engineered nanomaterials released from commercial nano-enabled products affect the morphology and behaviour of the zebrafish (D. rerio) embryo
Source: Ecotoxicology. 2026 Aug 20;35(7):154. doi: 10.1007/s10646-026-03139-z (PMC13493487; doi:10.1007/s10646-026-03139-z)
Supplement: Supplementary file 1 [file 10646_2026_3139_MOESM1_ESM.docx]

**Engineered nanomaterials released from commercial nano-enabled products affect the morphology and behaviour of the Zebra fish (*D. rerio*) embryo**

Mbuyiselwa Shadrack Moloi^1,2,3^, Raisibe Florence Lehutso^2^, Dana Kühnel^3,4^, Nils Klüver^3^, Mariana Erasmus^4^, Paul Johan Oberholster^1,2^, Melusi Thwala^1,5^

*^1^Centre for Environmental Management, University of the Free State, Bloemfontein*

*^2^Water Research Centre, Council for Scientific & Industrial Research, Pretoria*

*^3^Department of Ecotoxicology, Helmholtz Centre for Environmental Research, Leipzig*

*^4^Centre for Mineral Biogeochemistry, University of the Free State, Bloemfontein*

*^5^Science Advisory and Strategic Partnerships, Academy of Science of South Africa, Pretoria*

Supplementary data

Figure S1 (a). The specific morphological endpoints affected by exposure to SS1-nTiO_2._ Concentrations were prepared by diluting 100% (v/v) released media in a range of 0.94-60%; total Ti was quantified to be 0.79±0.04 mg/L in 100% media.

Figure S1 (b). The specific morphological endpoints affected by exposure to SS1-nTiO_2._ Concentrations were prepared by diluting 100% (v/v) released media in a range of 0.94 – 60%; total Ti was quantified to be 0.79±0.04 mg/L in 100% media.

Figure S2 (a). The specific morphological endpoints affected by exposure to SS2-nTiO_2._ Concentrations were prepared by diluting 100% (v/v) released media in a range of 0.94 – 7.5%; total Ti was quantified to be 0.17±0.03 mg/L in 100% media.

Figure S2 (b). The specific morphological endpoints affected by exposure to SS2-nTiO_2._ Concentrations were prepared by diluting 100% (v/v) released media in a range of 0.94 – 7.5%; total Ti was quantified to be 0.17±0.03 mg/L in 100% media.

Figure S3. The specific morphological endpoints affected by exposure to TC-nZnO. Concentrations were prepared by diluting 100% (v/v) released media in a range of 0.3 – 20%; total Zn was quantified to be 5.59±0.37 mg/L in 100% media.

Figure S4. The specific morphological endpoints affected by exposure to TC-Zn^2+^. Concentrations were prepared by diluting 100% (v/v) released media in a range of 0.3 – 20%; total Zn was quantified to be 9.08±0.79 mg/L in 100% media.

Figure S5. The specific morphological endpoints affected by exposure to SC-nAg. Concentrations were prepared by diluting 100% (v/v) released media in a range of 0.15 – 10%; total Ag and Ti were quantified to be 0.35±0.02 mg/L and 1.53±0.05 mg/L, respectively, in 100% media.

Figure S6. The specific morphological endpoints affected by exposure to SC-Ag^+^. Concentrations were prepared by diluting 100% (v/v) released media in a range of 0.15 – 10%; total Ag was quantified to be 0.10±0.005 mg/L in 100% media.
